# Exploring planar and nonplanar siligraphene: a first-principles study†

**DOI:** 10.1039/c9ra01037h

**Published:** 2019-04-17

**Authors:** Xudong Tang, Wenchao Liu, Chaobo Luo, Xiangyang Peng, Jianxin Zhong

**Affiliations:** Hunan Key Laboratory of Micro-Nano Energy Materials and Devices, School of Physics and Optoelectronics, Xiangtan University Xiangtan City Hunan Province 411105 P. R. China xiangyang_peng@xtu.edu.cn

## Abstract

Siligraphenes (g-SiC_*n*_ and g-Si_*n*_C) are a novel family of two dimensional materials derived from the hybrid of graphene and silicene, which are expected to have excellent properties and versatile applications. It is generally assumed that g-SiC_*n*_ is planar whereas g-Si_*n*_C is nonplanar. Based on first-principles calculations, we have explored the planarity and nonplanarity for g-SiC_*n*_ and g-Si_*n*_C (*n* = 3, 5, and 7). It is found that the silicene-like g-Si_5_C and g-Si_7_C, though buckled, are actually energetically quite close to their planar counterpart. We found a new high buckled g-Si_7_C, which is much more stable and looks disordered. g-SiC_7_, though accepted to be planar, is identified to be nonplanar in fact. We focused on the widely studied g-SiC_7_ to illustrate the difference induced by planarity and nonplanarity. The total energy calculation and phonon spectrum show that the nonplanar g-SiC_7_ is very energetically favorable and dynamically stable. The buckling leads to a considerable change in band structure, but the Dirac cones and the energy gap are still preserved. It is further found that g-SiC_7_ has valley-contrasting Berry curvatures, suggesting potential application of siligraphene in valleytronics. The planar and nonplanar g-SiC_7_ have quite similar lattice thermal properties, which are close to those of graphene. Our calculations indicate the importance of examination of the planarity and nonplanarity in the study of siligraphene.

## Abstract 2

New nonplanar g-SiC_7_ and g-Si_7_C have been found. g-Si_5_C, though buckled, is energetically very close to its planar counterpart.

## Graphical abstract

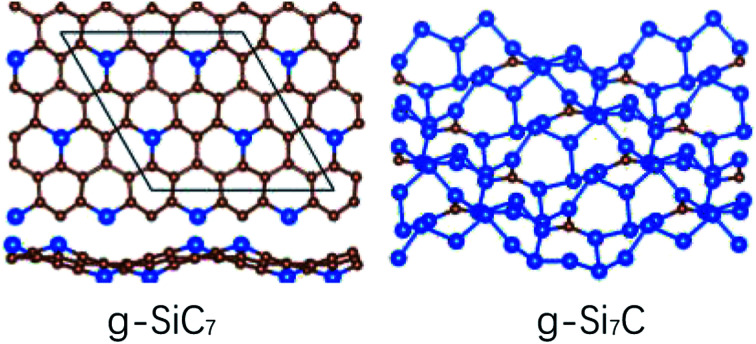

## 1. Introduction

Since its discovery, graphene has attracted much attention due to its unique properties and promising applications in future electronic devices.^1–3^ Silicene, a silicon version of graphene, has aroused new interest in silicon materials, not only because of its Dirac electron scattering spectrum at the Fermi level,^4,5^ but also because of its compatibility with the existing silicon-based electronic industry. However, graphene and silicene are gapless and hence not applicable for logic computing units. There has been a lot of effort towards the band gap opening,^6–9^ but achieving energy gaps in the range of 1.0–2.0 eV at room temperature is still challenging.

Recently, siligraphene, *i.e.*, Si-doped graphene, has emerged as a new 2D material. Siligraphenes have shown a variety of interesting properties. Depending on the stoichiometry and chemical bonding of siligraphene, the band gap changes from 0 to 2.87 eV. g-SiC_2_ is expected to be a solar cell material because it possesses a band gap of 1.09 eV.^10^ g-SiC_3_ is found to be a topological insulator,^11^ whereas g-SiC_5_ is a semi-metal with excellent properties for gas sensing.^12^ g-SiC_7_ has a great potential in photovoltaics with an energy gap (1.13 eV) desirably within the infrared and visible light range, in which it has superior light absorptance and solar energy conversion rate.^13^ After metallization, g-SiC_7_ becomes a high capacity hydrogen storage material.^14^ The electrical conductivity, thermal and optical properties of g-SiC_7_ are also investigated.^15,16^

Till now, on one hand, the studies of g-SiC_*n*_ (*n* > 1) are based solely on the planar configuration.^17^ The possible explanation is that the majority of the bonds are C–C bonds in g-SiC_*n*_ (*n* > 1), which do not favor buckling due to the supposed C–C sp^2^ hybridization. On the other hand, g-Si_*n*_C (*n* > 1) is generally believed to be nonplanar,^17^ because it has a majority of Si–Si bonds, which in silicene prefer sp^3^ hybridization and lead to a buckling of 0.45 Å.^18^ However, the buckled atomic structure of g-Si_*n*_C and the stability with respect to the planar structure has not been addressed. As known, Si–C bond is longer than C–C bond and shorter than Si–Si bond. To accommodate Si–C bonds in g-Si_*n*_C and g-SiC*_n_*, the hexagonal atom rings will be deformed. Whether such deformation will lead to buckling or not may depend on the concentration of Si doping, strain and the symmetry of the system. The difference induced by the planarity or nonplanarity in the properties of siligraphene also needs to be studied.

By performing first-principles calculations, we studied the atomic, electronic structures of siligraphenes. A variety of planar and nonplanar configurations have been considered and their relative stability has been compared. We found new nonplanar structures and discussed the differences induced by planarity and nonplanarity. In particular, g-SiC_7_ is of great interest recently and has been investigated in the context of photovoltaics, hydrogen storage, electrical, thermal and optical properties.^13–16^ We studied SiC_7_ in more detail to evaluate the stability of the planar and nonplanar structures and reveal the electronic, thermal and the Berry phase related properties. The new insights into siligraphene regarding the planarity and nonplanarity will be helpful for future study of the physical properties of group IV atom doped graphene, silicene and germanene.

## 2. Methods

The first-principles calculations were carried out by using VASP^19^ (Vienna ab initio simulation package) based on density functional theory (DFT). The generalized gradient approximation (GGA-PBE)^20^ is employed. The energy cutoff for plane wave expansion is set to be 520 eV. The siligraphene monolayer is modelled by a slab in a supercell with a vacuum of 18 Å. The *k*-mesh to sample the first Brillouin zone of the primitive cell is a Γ centered 8 × 8 × 1 grid. Atomic relaxation is done until the force on each atom is less than 10^−3^ eV Å^−1^. The lattice is also simultaneously relaxed. In the following, we mean relaxation implicitly as simultaneous atomic and lattice relaxation.

The vibrational and lattice thermal properties are studied by using the DFPT (density functional perturbation theory) package PHONOPY.^21,22^ To obtain the second-order harmonic interatomic force constants, a supercell containing 128 atoms is employed. The thermodynamic properties are calculated using a 21 × 21 × 1 **q** grid.

## 3. Results and discussion

We first calculated graphene, silicene and g-SiC as a reference. It is found that graphene is completely flat^23^ whereas silicene has a buckling of 0.44 Å.^18^ The C–C and Si–Si bond lengths in graphene and silicene are 1.420 and 2.276 Å, respectively, agreeing well with the previous studies.^24–26^ For g-SiC, we tried many initial nonplanar structures and all of them converge to a planar g-SiC after relaxation of the atomic position and the lattice. The calculated Si–C bond length (1.785 Å), lattice constant (*a* = *b* = 3.092 Å) and band structure are in good agreement with previous calculations.^26,27^

In the following study of siligraphene, the total energy of the optimized flat structure is obtained first for later comparison. To explore the nonplanar siligraphenes, a lot of initial buckled structures are considered and relaxed to see if they finally lead to a nonplanar or a planar structure. To allow more freedom to displace Si or C atoms, we use a 2 × 2 unit cell in addition to the primitive cell of siligraphene when necessary. If the final relaxed structure is planar, then the buckled structure cannot be stabilized. If eventually a nonplanar structure is stabilized, we compare the total energy of the planar and nonplanar structures to determine which one is more stable.

### 3.1 g-Si_3_C, Si_5_C and Si_7_C

Although it is supposed that g-Si_*n*_C has a buckled structure,^17^ the nonplanar structures have not been discussed yet. In particular, the stability of the buckled structure with respect to the planar one has not been studied. We investigated and compared the planar and nonplanar structures of g-Si_*n*_C in more detail. The initial nonplanar structures for g-Si_*n*_C are obtained by substituting some of the Si atoms in the buckled silicene by C. Besides, we also tried other initial nonplanar structures by randomly displacing the C and Si atoms. The results are listed in Table 1.

**Table 1:** The lattice parameters *a* and *b*, C–C and Si–C bond lengths and buckling *h* of the optimized g-SiC_*n*_ and g-Si_*n*_C (*n* = 3, 5, 7)^a^

Structures	Bonds length (Å)			
	*a* = *b* (Å)	C–C	Si–C	*h* (Å)
2 × 2 flat-SiC_7_	10.579	1.438/1.550	1.691	0
2 × 2 buckled-SiC_7_	10.296	1.435/1.513	1.753	1.7
Flat-SiC_5_	4.652	1.453	1.764	0
Flat-SiC_3_	5.624	1.439	1.813	0
2 × 2 high buckled-Si_7_C	13.250	2.322/2.283	1.812	3.77
2 × 2 flat-Si_7_C	14.768	2.259/2.179	1.863	0
Low buckled-Si_7_C	7.339	2.282/2.197	1.868	0.491
Flat-Si_7_C	7.385	2.258/2.179	1.863	0
Low buckled-Si_5_C	6.258	2.262	1.809	0.332
Flat-Si_5_C	6.279	2.250	1.806	0
Flat-Si_3_C	7.038	2.254	1.809	0

a The low buckled g-Si_5_C and Si_7_C indicate the silicene-like low buckled structure.

**Fig. 1:**
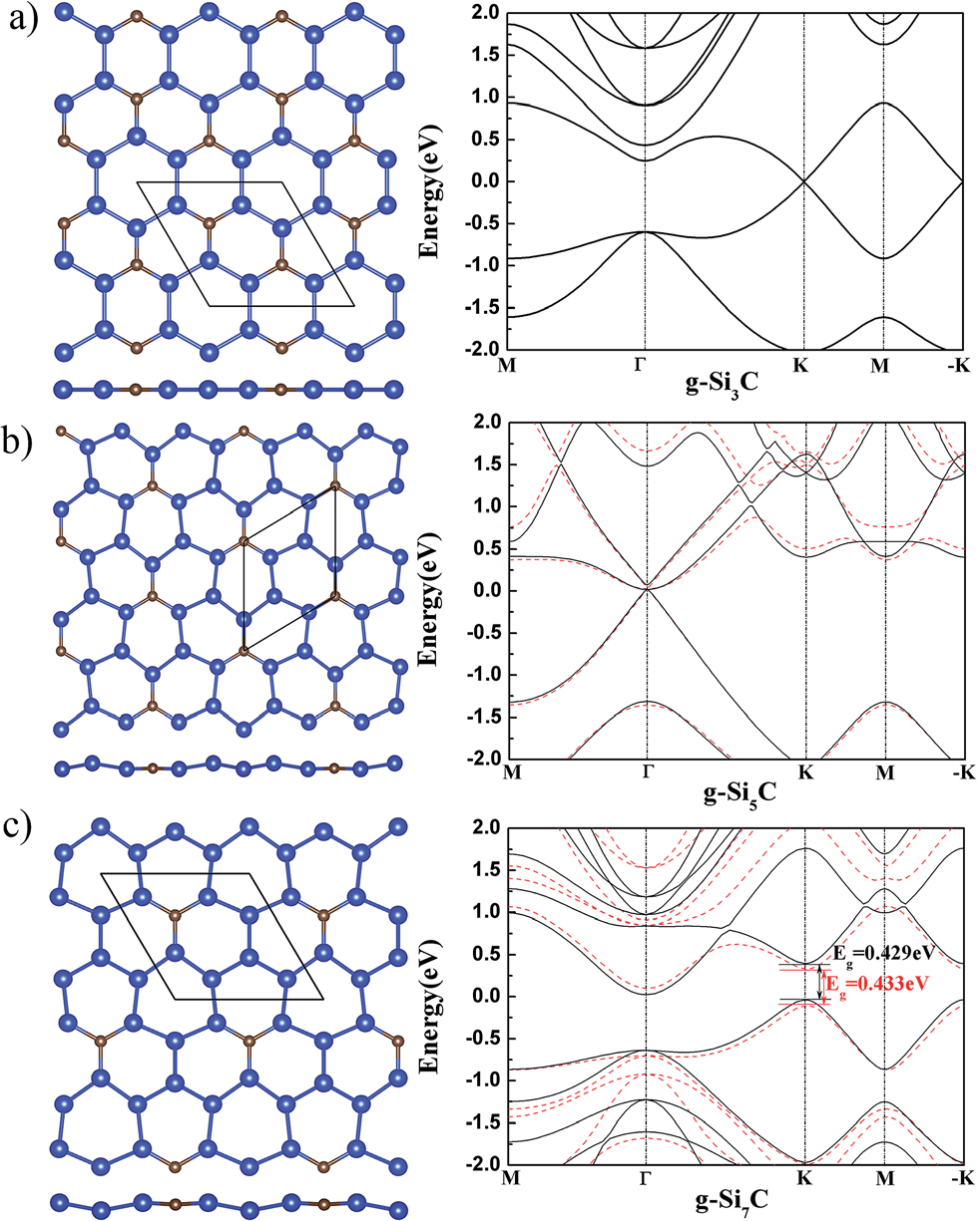
The atomic structure (left) and band structure (right) of g-Si_*n*_C for *n* = 3 (a), 5 (b) and 7 (c). In the left panel, the top (upper) and side (lower) views are shown. The smaller brown and larger blue spheres stand for the C and Si atoms, respectively. The diamond designates the primitive cell. In the right panel, the black solid and red dashed curves denote the band structure of the flat and buckled structure, respectively.

It might be first expected that g-Si_3_C, being closer to silicene than to graphene in stoichiometry and having Si–Si bonds, should have a buckled structure.^17^ However, after relaxation of atomic position and lattice, it is found that all the tried initial buckled structures are finally turned to be flat,^11^ as shown in Fig. 1(a). The band has gapless Dirac cone at K and −K points, in agreement with previous study.^11^

The g-Si_5_C has higher Si concentration than g-Si_3_C. After relaxation of the initial buckled structures, it is found that the Si atoms are buckled up and down around the C atom with a Si–Si buckling of 0.33 Å [see Fig. 1(b)]. The lattice constant is reduced by 0.021 Å with respect to that of flat g-Si_5_C. It is found that the buckled g-Si_5_C is only 3.7 meV per atom lower in energy than its flat structure, whereas the energy gain due to buckling in silicene is 30 meV per atom.^18^ Therefore, the stability of the buckled structure is much reduced by the incorporated C atoms, though the concentration of C atoms is low. In comparison with the energy difference of AA and AB stacked bilayer graphene (20 meV per atom by DFT and 6.2 meV per atom by Monte Carlo),^28,29^ which have both been experimentally observed,^29,30^ it can be expected that planar and nonplanar structures for g-Si_5_C, being closer in energy, may coexist at room temperature. The electronic bands of the nonplanar and flat g-Si_5_C almost overlap near the Dirac cone around Γ point but are more different at other *k* points and energy range [see Fig. 1(b)]. There is no energy gap overall, but the Dirac cone has a gap of 0.06 eV.

We also obtained a silicene-like buckled g-Si_7_C with a Si–Si buckling of 0.491 Å after relaxation, as shown in Fig. 1(c). This buckled g-Si_7_C is lower in energy than the planar one by 8.8 meV per atom, though larger than the corresponding value of 3.67 meV per atom for g-Si_5_C, the stability of the nonplanar structure with respect to the planar one is still significantly reduced in comparison with silicene. The energy bands difference between the flat and buckled g-Si_7_C is more apparent than that for g-Si_5_C. There is an energy gap of 0.433 eV at the Dirac point of the buckled g-Si_7_C.

**Fig. 2:**
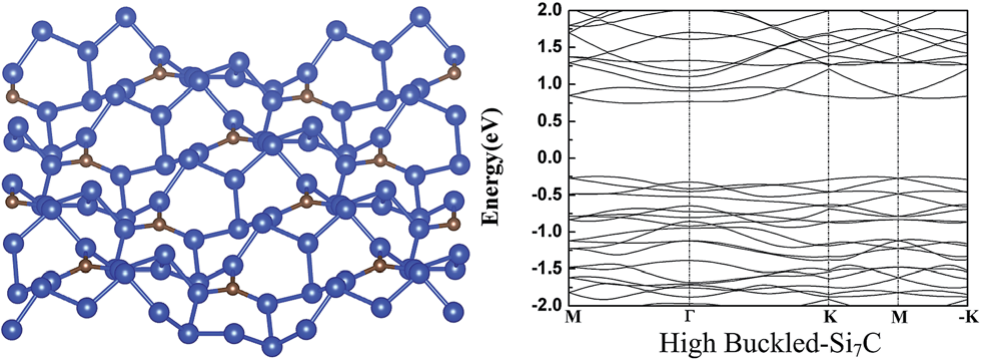
The atomic (left) and band (right) structure of the high buckled-Si_7_C. For legend, see caption of Fig. 1.

We also explored other possible initial buckled structures for g-Si_7_C. After atomic and lattice relaxation, a very stable high buckled 2 × 2 g-Si_7_C is found (see Fig. 2), which looks disordered and very different from the low buckled g-Si_5_C and Si_7_C discussed above. The Si–Si buckling is as large as 3.77 Å and the total energy is significantly lower than that of the planar g-Si_7_C by 71.3 meV per atom. Due to the large buckling, the lattice constant is decreased by 1.52 Å with respect to that of the flat 2 × 2 g-Si_7_C. It is surprising that a small amount of C atoms can give rise to such a large structural change. We made exploration of different buckled structures for g-Si_3_C and Si_5_C in a similar way, and did not find similar disordered buckled structures. It might be assumed that with the decrease of the concentration of C atoms, the structure will be reduced in complexity and will converge to that of silicene. Our calculations show that at certain range of C concentration, this trend does not apply. The electronic structure is significantly changed. The Dirac cones are destroyed and the gap is increased to 0.98 eV, as shown in Fig. 2.

### 3.2 g-SiC_3_, SiC_5_ and g-SiC_7_

**Fig. 3:**
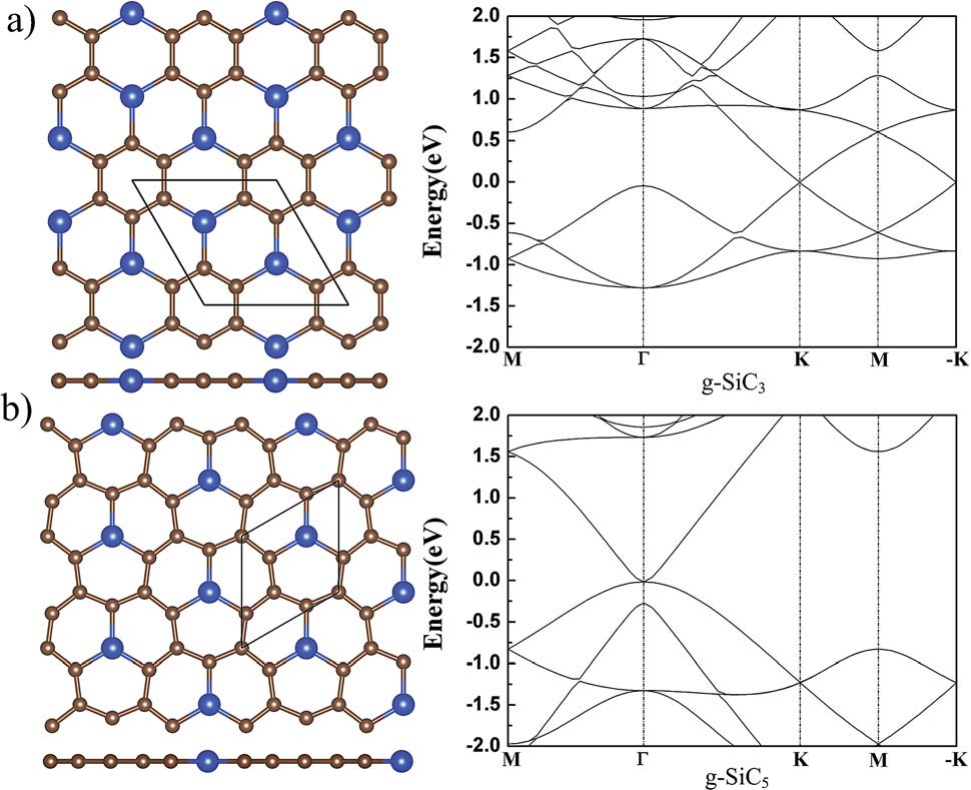
The atomic (left) and band (right) structure of g-SiC_3_ and SiC_5_. The top (upper) and side (lower) views of the atomic structure are shown in the left panel. For legend, see caption of Fig. 1.

There are more C atoms than Si atoms in g-SiC_3_ and SiC_5_. Various initial nonplanar structures have been tried and relaxed, and all end up with a flat g-SiC_3_ and SiC_5_ after relaxation. Therefore, g-SiC_3_, and SiC_5_ can exist only in planar form. The atomic and electronic structures agree with the previous calculation,^31^ which are shown in Fig. 3 for completeness.

**Fig. 4:**
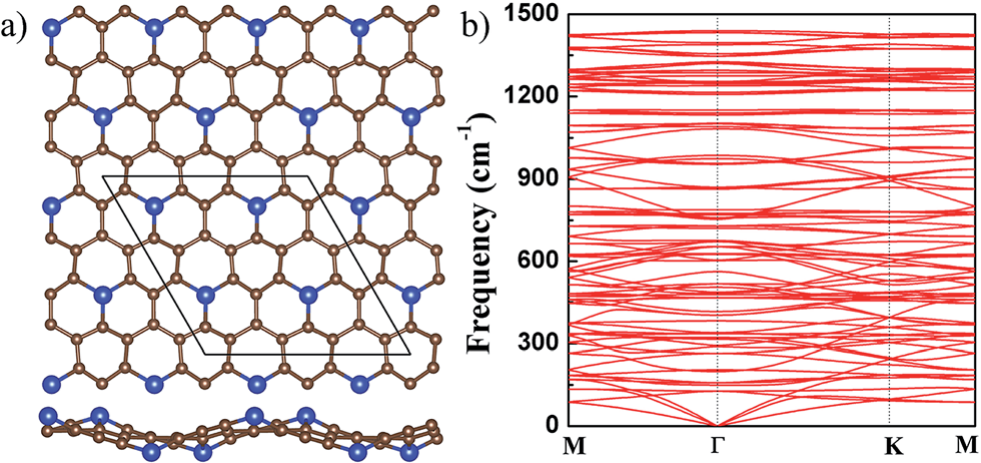
(a) The top (upper) and side (lower) views of the buckled g-SiC_7_. For legend, see caption of Fig. 1. (b) The phonon spectrum of the buckled g-SiC_7_.

In g-SiC_7_, the concentration of Si is only 12.5%. With the reduction of Si concentration, one may expect that g-SiC_7_ should be flat, as its stoichiometry is further approaching to that of graphene.^17,31^ We started from an initial flat structure and found that the system remains to be flat after relaxation. Although there are pure C hexagonal rings, three C atoms in the C ring are each bonded to a Si atom and the other three C atoms are each connected to a C atom, making the C ring irregular and deformed, as shown in Fig. 4(a). In the relaxed flat g-SiC_7_, the Si–C bond length is 1.691 Å and C–C bond length varies from 1.438 to 1.550 Å (see Table 1). As mentioned above, g-SiC_3_ (g-SiC_5_) only has flat configuration, in which all the Si–C and C–C bonds are 1.813 (1.766) Å and 1.439 (1.455) Å, respectively, as shown in Table 1.

Comparing with the Si–C bond length 1.785 Å in g-SiC, one can see that the Si–C bond is considerably more compressed in flat g-SiC_7_. Since compressive strain usually increases the tendency of the out-of-plane buckling, it is more likely for g-SiC_7_ to exist in a nonplanar form.

It is found that if the Si atoms are displaced alternately up and down in the initial 2 × 2 g-SiC_7_ structure, a nonplanar g-SiC_7_ is finally stabilized with Si atoms alternately up and down, which is significantly lower in energy than the flat g-SiC_7_ by 21 meV per atom. The buckling between the up and down Si atoms, as shown in Fig. 4(a), is as large as 1.7 Å. The optimized lattice constant of buckled g-SiC_7_ is 10.296 Å, which is 0.283 Å smaller than that of the flat 2 × 2 g-SiC_7_ as a result of the large buckling. The Si–C bond length is considerably increased from 1.691 to 1.753 Å and the longest C–C bond length is reduced from 1.550 to 1.513 Å, effectively releasing the stress. We further studied the dynamical stability of the buckled g-SiC_7_ by calculating the phonon spectrum, as shown in Fig. 4(b). It can be seen that there is no imaginary frequency, indicating that the buckled g-SiC_7_ is dynamically stable. We also considered two different Si distributions in g-SiC_7_ (see Fig. S1 in ESI†), and also found that the nonplanar structures are more stable.

In the following, we will focus on this nonplanar g-SiC_7_ and study its electronic, thermal, and valleytronic properties.

**Fig. 5:**
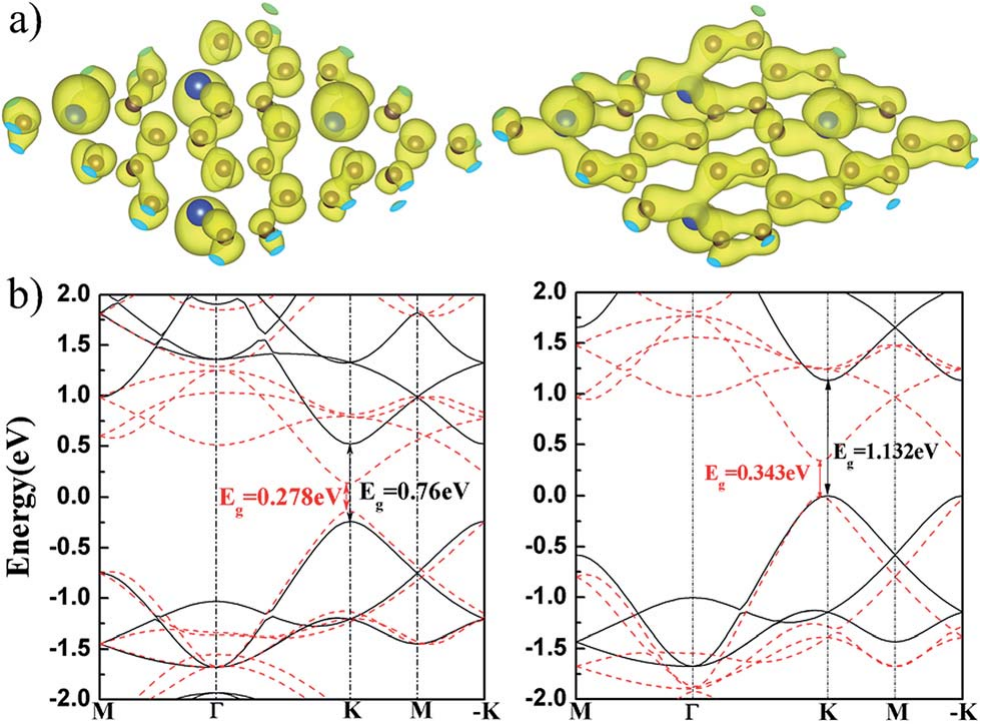
(a) The charge distribution of CBM (left) and VBM (right) at K point of the buckled g-SiC_7_; (b) the GGA-PBE (left) and HSE06 (right) band structures of the flat (black solid line) and buckled (red dashed line) g-SiC_7_.

We calculated the charge density of the valence band maximum (VBM) and conduction band minimum (CBM) at K point, as show in Fig. 5(a). It is found that the VBM and CBM states are mainly contributed by p_*z*_ orbital of Si and C. The charge overlapping in VBM states is larger than that in CBM because their p_*z*_ states are in bonding and anti-bonding hybridization, respectively. The electronic band structures of flat and buckled g-SiC_7_ layer are also calculated. It can be seen in Fig. 5(b) that the band gap of the flat SiC_7_ occurs at the K and −K point with a magnitude of 0.76 eV (GGA-PBE) and 1.132 eV (HSE06 (ref. 32 and 33)), in good accord with the previous calculations.^13^ The gap of the nonplanar g-SiC_7_ is much reduced to 0.278 eV (GGA-PBE) and 0.343 eV (HSE06), which is still in the infrared energy range. In addition, the position of the gap is shifted a bit away from the K and −K point. Both flat and buckled SiC_7_ are direct band gap semiconductors.

**Fig. 6:**
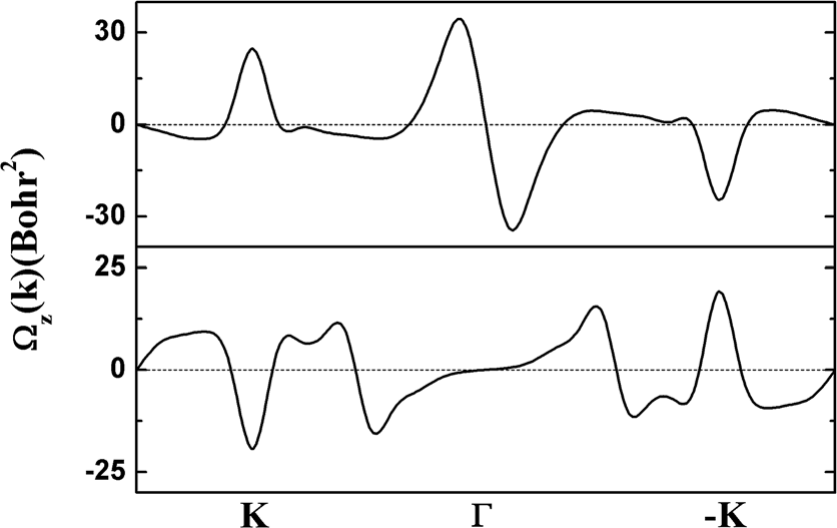
The calculated Berry curvature of the highest valence band (upper) and the lowest conduction band (lower) of g-SiC_7_.

The electronic structure of g-SiC_7_ suggests another important application for graphene and silicene, namely valleytronics, which requires gap opening. In comparison with graphene and silicene, the advantage of g-SiC_7_ and many other siligraphenes is that they already have a considerable native gap at the Dirac cones (valleys).^13,17,31^ We calculated the Berry curvature of the highest valence band and the lowest conduction band, using the Kubo formula.^34^ As depicted in Fig. 6, the Berry curvature is opposite at *K* and −*K*, *i.e. Ω*_z_ (−*K*) = −*Ω*_z_ (*K*), which underlies the Berry phase related physics, such as valley Hall effect.^35^ Our calculations indicate that siligraphene has potential applications in valleytronics.

We computed the lattice thermodynamic properties of the buckled g-SiC_7_. As detailed in ref. 22, the phononic entropy *S*_ph_, Helmholtz free energy *F*_ph_ and constant volume heat capacity *C*_V,ph_ are calculated based on the following formulae:1
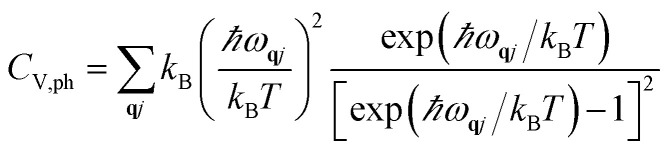
2
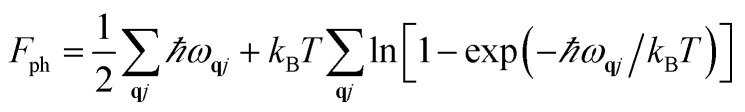
3
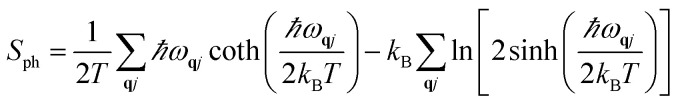
where *ω*_**q***j*_ is the phonon frequency with **q** and *j* being the wave vector and band index, respectively. The total Helmholtz free energy *F* can be approximated by^22^

**Fig. 7:**
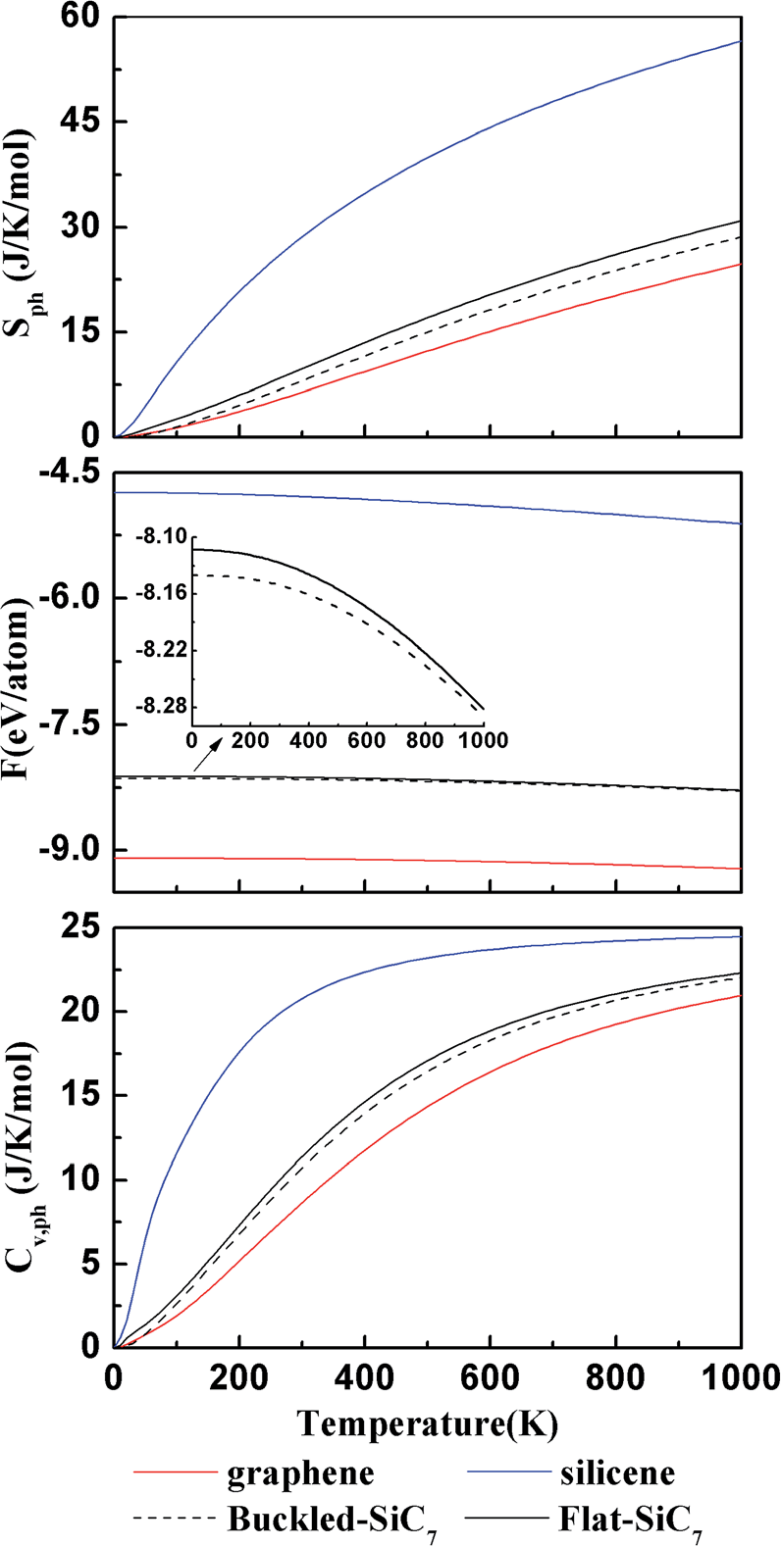
The calculated entropy *S*_ph_ (upper), total Helmholtz free energy *F* (middle), and constant volume heat capacity *C*_V,ph_ (bottom) for graphene, silicene, buckled g-SiC_7_ and flat g-SiC_7_. The inset in the middle panel is a zoomed view of the total Helmholtz free energy of the buckled g-SiC_7_ and flat g-SiC_7_.

4 *F* ≈ *U*_el_ + *F*_ph_

where *U*_el_ is total energy of electronic structure from the first principles calculation. For comparison, we also calculated the corresponding thermodynamic properties of graphene,^36–38^ silicene and flat g-SiC_7_, as shown in Fig. 7. It can be seen that the curves of the buckled g-SiC_7_ are between those of graphene and silicene and basically follow the same trend. From 0 to 1000 K, the entropy of g-SiC_7_ grows almost linearly with temperature. The free energy decreases relatively slowly with temperature below 400 K but quickly above 400 K. The heat capacity has a linear growth below 500 K and tends to saturate near 1000 K. g-SiC_7_ is much more stoichiometrically closer to graphene and accordingly the curves of g-SiC_7_ are much closer to those of graphene. The entropy and constant volume heat capacity of the buckled g-SiC_7_ are a little lower than those of flat g-SiC_7_. The buckled g-SiC_7_ has a lower total Helmholtz free energy than the flat g-SiC_7_ up to 1000 K, indicating that the former is more thermodynamically stable than the latter.

## 4. Conclusion

In summary, siligraphenes g-SiC_*n*_ and g-Si_*n*_C with *n* = 3, 5, 7 have been investigated by first-principles calculation. Both planar and nonplanar configurations have been explored and compared. It is found that although g-Si_5_C and Si_7_C have nonplanar silicene-like structure, they are quite energetically close to their planar counterparts. For g-Si_7_C, we found a new disordered nonplanar structure with large buckling, which is far more energetically favorable than the planar and low buckled g-Si_7_C. g-SiC_7_, though assumed to be planar in previous studies,^17^ has a much more stable nonplanar structure. We studied g-SiC_7_ in more detail to show the effect of planarity and nonplanarity. It is found that the electronic structure is appreciably affected with the gap reduced from 0.76 (1.132 for HSE06) to 0.278 (0.343 for HSE06) eV. The Dirac valleys are preserved and the valley contrasting Berry curvature suggests potential applications in valleytronics. The lattice thermal properties of planar and nonplanar g-SiC_7_ are quite similar and both are close to those of graphene. Our studies suggest that both planarity and nonplanarity should be scrutinized in the first place when study siligraphene and other group IV atom doped graphene, silicene and germanene.

## Conflicts of interest

There are no conflicts of interest to declare.

## Supplementary Material

RA-009-C9RA01037H-s001

RA-009-C9RA01037H-s002

RA-009-C9RA01037H-s003
