# Supplementary material for: Exploring planar and nonplanar siligraphene: a first-principles study
Source: RSC Adv. 2019 Apr 17;9(22):12276–81. doi: 10.1039/c9ra01037h (PMC9063529; doi:10.1039/c9ra01037h)
Supplement: RA-009-C9RA01037H-s003 [file RA-009-C9RA01037H-s003.pdf]

Electronic Supplementary Material (ESI) for RSC Advances.
This journal is © The Royal Society of Chemistry 2019

Supplementary Information for
"Explore the planar and nonplanar siligraphene: a first-principles study"

We studied two additional Si distributions in 2×2 g-SiC_7 as depicted in Fig. S1. For each Si distribution, planar and nonplanar structures are calculated. It is found that the lattice of planar structure has appreciably larger lattice constants than the corresponding nonplanar one. The Si atoms in the nonplanar structure are buckled in an alternately up and down fashion. The nonplanar structure is considerably more stable than its corresponding planar structure. The nonplanar structures shown in Fig. S1 (a) and (b) are 19.68 and 178.5 meV/atom lower in energy than their planar counterparts. The Si-Si bonds in g-SiC_7 shown in Fig. S1(b) have very high tendency to be buckled. The lattice constants, bondlengths and the height of buckling are listed in Table S1.

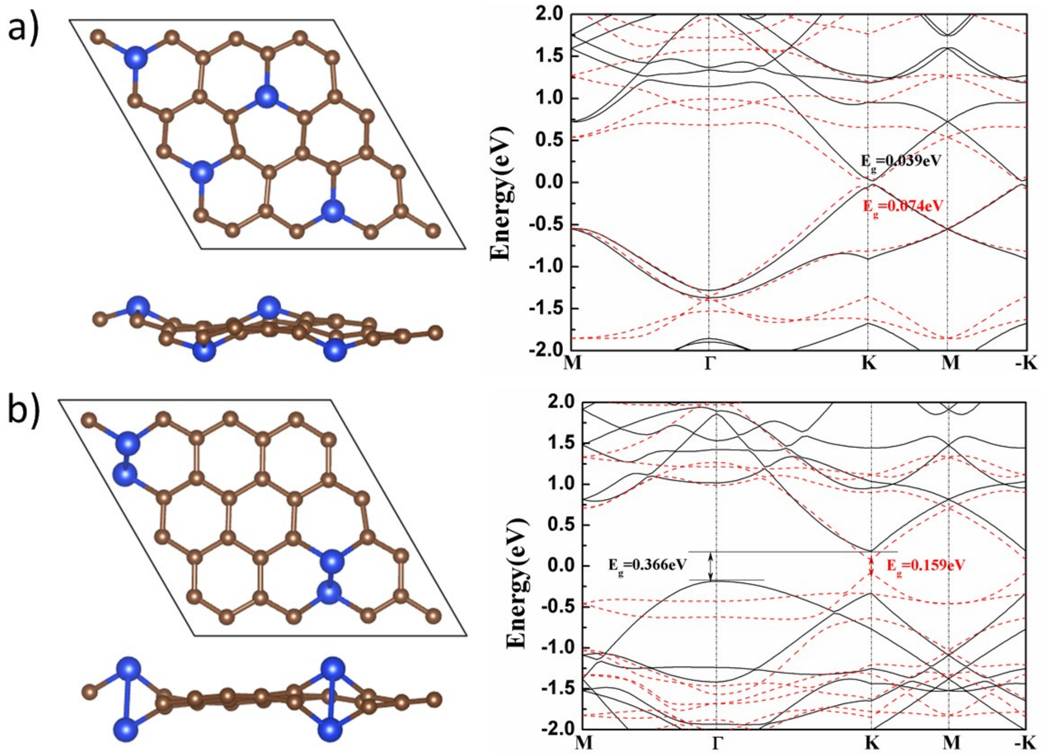

Fig. S1 (a) and (b) the top and side views (left panel) and band structure (red dashed line in the right panel) of two different distributions of Si atoms in buckled 2×2 g-SiC_7. The band structure of the corresponding planar structure is also presented (black line in the right panel) for comparison. The small brown and big blue spheres denote the C and Si atoms. The diamond indicates the 2×2 unitcell.

Table S1 The lattice parameters, C-C and Si-C bond lengths and buckling h of the optimized g-SiC_7 with Si distributions as shown in Fig. S1. The letters a and b in the first column correspond the structures shown in Fig. S1 (a) and (b), respectively.

		Bonds length(Å)		
Structure	a=b(Å)	C-C	Si-C	h(Å)
a-g-SiC_7-flat	10.589	1.466/1.577	1.719	0
a-g-SiC_7-buckled	10.348	1.411/1.513	1.757	1.66
b-g-SiC_7- flat	10.669	1.416/1.494	1.733	0
b-g-SiC_7- buckled	10.209	1.443/1.495	1.919	2.24
